# Assessment of equity and efficiency of magnetic resonance imaging services in Henan Province, China

**DOI:** 10.1186/s12962-023-00440-0

**Published:** 2023-05-23

**Authors:** Xiaoling Huang, Yan Wei, Hui Sun, Jiaqi Huang, Yingyao Chen, Jingliang Cheng

**Affiliations:** 1 National Health Commission Key Laboratory of Health Technology Assessment, School of Public Health, Fudan University, Shanghai, 200032 P. R. China; 2 School of Public Health, Fudan University, Shanghai, China; 3 WHO Collaborating Center for HTA and Management, Shanghai, China; 4 Department of magnetic resonance, The First Affiliated Hospital of Zhengzhou University, Zhengzhou, China

**Keywords:** Magnetic resonance imaging (MRI), Equity, Gini coefficient, Agglomeration, Efficiency, Data envelopment analysis (DEA)

## Abstract

**Background:**

By evaluating equity and effectiveness, this study provides evidence-based knowledge for scientific decision-making and the optimization of magnetic resonance imaging (MRI) configuration and utilization at the provincial level.

**Methods:**

Using data from 2017, we applied a Gini coefficient to analyze the equity of MRI services in 11 sample cities in Henan province. An agglomeration degree was then applied to measure equity from the perspective of population and geography, and a data envelopment analysis was used to evaluate MRI efficiency.

**Results:**

The overall Gini coefficient of MRI allocation by population in the 11 sample cities is 0.117; however, equity varies considerably among the sample cities. The sample’s comprehensive efficiency is only 0.732, indicating the overall ineffectiveness of provincial MRI utilization. The pure technical and scale efficiencies of four sample cities are below 1, indicating lower MRI effectiveness than the rest.

**Conclusions:**

Although the overall equity of configuration at the provincial level is relatively good, equity varies at the municipal level. Our results demonstrate a low MRI utilization efficiency; accordingly, policymakers should dynamically adjust the policy based on equity and efficiency.

## Background

In China, large medical equipment refers to either medical equipment included in the management list of the Health Department under the State Council or equipment not included in the list but priced above 5 million Chinese yuan (CNY) and allocated at provincial level hospitals for the first time.

In 2004, the Ministry of Health (MoH) released the “Management Methods on Allocation and Utilization of Large Medical Equipment” [1], and in 2005, the National Health and Family Planning Commission (NHFPC; formerly the MoH) released the “Guiding Opinions on National Class B Large Medical Equipment Allocation Planning” [2]. Since then, the management of large medical equipment configurations in China has been implemented under the National Health Commission (NHC, formerly the NHFPC).

As part of the ‘‘Regulations for the Management of Configuration and Use of Large Medical Equipment,” the Certificate of Need (CON) policy, established as the list went into effect, aims to improve “appropriate allocation and the efficient use of medical equipment” through regional health planning and quota control. The National Health Commission controls the total amount of large medical equipment in China, and provincial health departments formulate allocation plans under the NHC quota [3]. The NHC is responsible for the CON licensure of Class A large medical equipment, whereas provincial health departments are responsible for Class B large medical equipment. Hospitals must apply for CON before purchasing large medical equipment.

In the past 20 years, the CON policy has positively influenced rational configuration, clinical application, cost control, and health improvement in China and initially ensured the fair allocation of health resources. However, owing to various early-stage factors, blind configuration of equipment led to two rounds of strict large medical equipment configuration planning in 2009 and 2011 [4], which brought about corresponding management difficulties in the next few years: First, the purchasing demand from hospitals could not be met owing to strict quota limitations, which has led the supply–demand conflict to become increasingly prominent. Second, owing to low patient volume, limited technical personnel and equipment, secondary hospitals have limited large medical equipment utility. In contrast, tertiary hospitals often have more large medical equipment because of their large patient volume and strong scientific research capabilities, resulting in patients still being concentrated in tertiary hospitals; thus, the large medical equipment in tertiary hospitals is further overloaded [4].

Appropriate allocation and the efficient use of medical equipment can be measured by “equity” and “efficiency,” which are two value goals and measurement standards that need to be considered when assessing the allocation of medical resources [5–7]. Therefore, over the years, the government intervention mechanism in the management of large medical equipment configurations in China has been presenting new characteristics and trends through constant self-adjustment and correction: First, management is becoming increasingly refined, and the management system is moving from the initial extensive framework-type management toward more refined management that is more in line with economic development [8, 9]. Second, to achieve the goal of optimizing the allocation of health resources, both equity and efficiency are increasingly emphasized in configuration, that is, “equity first while considering efficiency,” so as to ensure that residents in all regions and residents at different income levels have equal rights to enjoy large medical equipment services while maintaining cost control and a focus on equipment utilization efficiency.

Many studies have been conducted in China on the implementation of large medical equipment configuration management policies in terms of equity, usage, and effectiveness; however, these studies are mostly limited to individual points such as equity or efficiency [10–18]. The Gini coefficient, Lorenz curve, and Theil index are applied to evaluate the configuration equity. Data envelopment analysis (DEA) and rank-sum ratio (RSR) analysis are widely used to assess efficiency [15, 16]. These studies do not reflect the situation of equity and efficiency of large medical equipment simultaneously in a comprehensive way, and fail to provide targeted improvement suggestions to align with the principle of “equity first while considering efficiency.” Some international studies on the management of large medical equipment are based on comparative analyses of one or several countries, focusing mainly on configuration status, utilization status, and management policies [17–19]. Developed and developing countries represented by the OECD have relatively high levels of MRI configuration and can well meet people’s needs. In-depth studies on large medical equipment service, demand, and configuration planning are relatively insufficient because of the advanced medical technology and well-designed service and management system in OECD countries.

Magnetic resonance imaging (MRI) is a regulated Class B large medical equipment, which is widely used in clinical practice. Its configuration has increased from 1.28 units per million people in 2009 to 5.02 units in 2017 [20]. This study used MRI as the research object and selected Henan province in China, which is close to a population of 10 million, as the case province. Using the framework of health service system evaluation and policy evaluation, this study chose two evaluation dimensions, equity and efficiency, to conduct an in-depth assessment based on the principle of “equity first while considering efficiency” in the policy of large medical equipment configuration management. The Gini coefficient and DEA were used to evaluate the equity and efficiency of MRI. This is the first study to apply matrix analysis to further analyze the configuration and utilization efficiency of MRI in different cities in Henan province based on previous analysis methods. This is done to understand the problems in configuration and efficiency that exist in different cities in Henan province and explore resource allocation that prioritizes equity and considers efficiency.

With the development of the social economy and people's demand for health, the number of large medical equipment is bound to increase. With the continuous improvement of the management policies for allocating large medical equipment, the management will become more refined. Therefore, it is necessary to consider the rationality of resource allocation from more dimensions. This study breaks through the singularity of previous studies and conducts a two-dimensional analysis of fairness and efficiency in the same region and period from the perspective of “equity first while considering efficiency.” Based on the research status of MRI equipment configuration in different cities in Henan province, this study provides recommendations for optimizing the provincial-level MRI equipment configuration policies. The methodology, research framework, and research tools used in this study can be applied to evaluate the allocation of other large medical equipment and health resources.

## Methods

**Fig.1:**
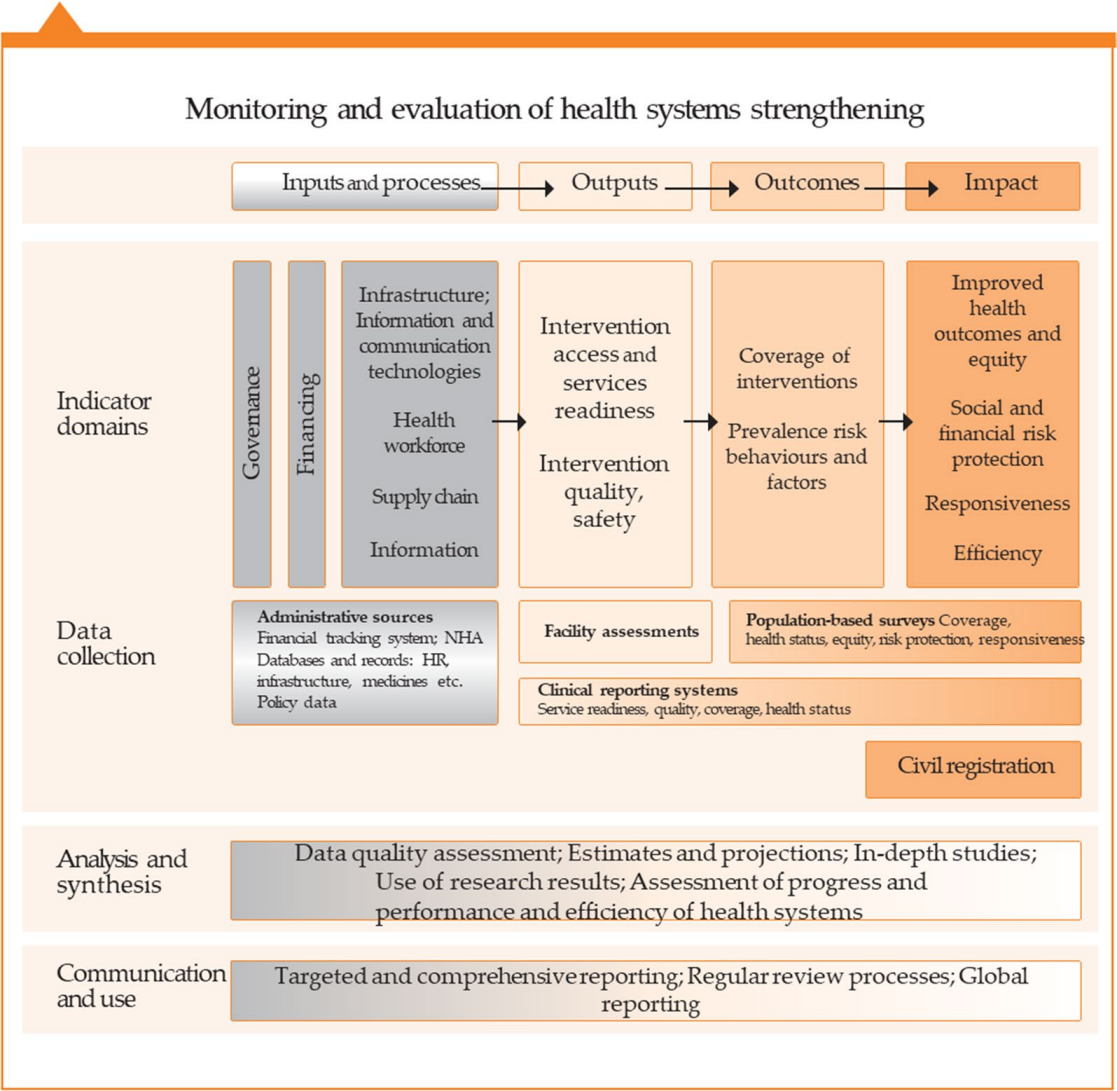
Monitoring and evaluation of health system strengthening

Based on the principle of rational allocation and effective use of large medical equipment, ensuring medical quality and safety, and controlling the rapid increase in medical expenses, this study drew on the World Health Organization’s health systems framework and monitoring and evaluation framework (WHO: MONITORING THE BUILDING BLOCKS OF HEALTH SYSTEMS) [21]. It also used Dunn’s six criteria for policy evaluation as a reference, namely, “effectiveness,” “efficiency,” “adequacy,” “equity,” “responsiveness,” and “appropriateness.” Two indicators of impact—equity and efficiency—were selected to assess the configuration and utilization of MRI in Henan province [22] Fig. 1.

### Sample and data collection

There are 18 prefecture-level cities in Henan province, with 586 MRI facilities among them. As the data on MRI utilization were not available for all cities, we selected 11 prefecture-level cities as our research sample. The medical facilities in these cities have 417 MRI machines. The number of MRI in Henan province was obtained through a questionnaire address to the provincial health department at the end of 2017. We obtained population data of the 11 sample cities from the 2018 Statistical Yearbook of Henan. For the efficiency analysis, data on the hospitals’ MRI utilization efficiency were obtained from the survey data of the project “MRI Service System Evaluation,” conducted in 2018. We also collected efficiency-related data from 78 MRI machines in the 11 sample cities through convenience sampling and included the data in our model. All data were collected from the participating hospitals via an online questionnaire. The questionnaire was designed according to the research purpose of the project “MRI Service System Evaluation.” It is a long questionnaire comprising seven parts with a total of 134 questions, including all MRI service-related information, such as information on the hospital, MRI department and staff, MRI configuration, MRI charging, MRI maintenance MRI manufacturer, and MRI service and utilization. A preliminary investigation was conducted on three hospitals before the questionnaire was finalized, following which the surveyed hospitals were trained on how to fill the questionnaire. The online questionnaire was disseminated among the participating hospitals for completion. Each participating hospital provided the required information through their respective accounts, assigning a dedicated individual to oversee the completion and verification of the results. The online questionnaire had to be submitted within 2 weeks from the date of activation.

The researchers were trained on the quality control standards for data collection. Each data collection team comprised two researchers, with one responsible for the initial review of completed questionnaires and the other for second-tier verification. Any questionnaire not meeting the specified criteria was rejected and returned to the hospital for revision. The research team contacted the hospital representative to ensure comprehensive understanding of the requirements before the resubmission process. Questionnaires that contained satisfactory responses to all queries were subjected to a rigorous two-tier verification process before being deemed eligible for further analysis. Those that did not meet the specified criteria, that is, lower than 80% completion or logical conflict within different questionnaire sections, were expunged from the database.

## Statistical analysis

### Equity assessment

**Fig.2:**
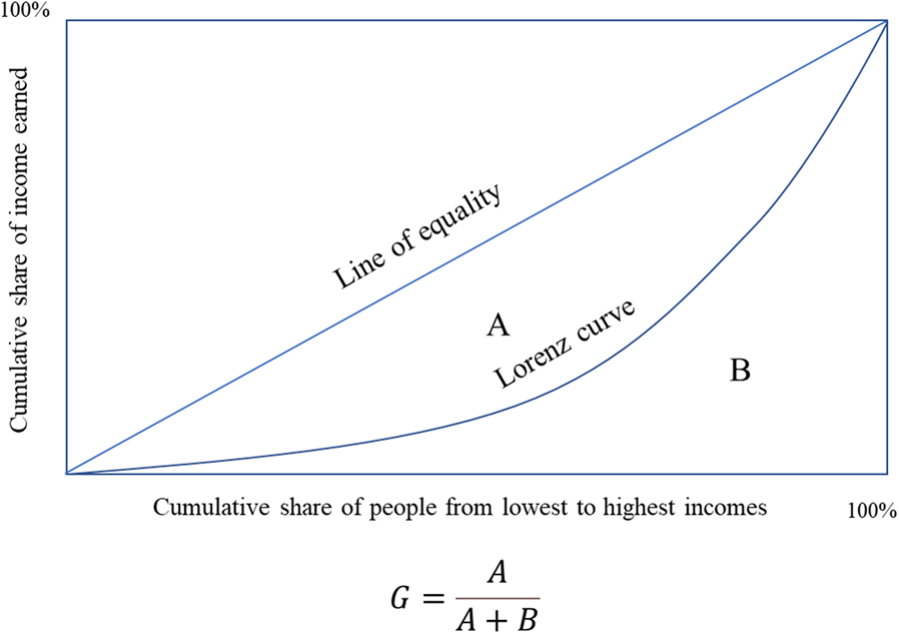
Lorenz curve

Conventionally, the Lorenz curve is used to reflect the degree of fairness in social income or property distribution and the fairness of health resource allocation. The Gini coefficient is often used as a quantitative index to reflect the fairness of social wealth distribution. This coefficient equals the ratio of the area enclosed by the absolute equity line, while the Lorenz curve is the area of the right triangle under the absolute equity line [23] Fig. 2.

A Gini coefficient is calculated as follows:

1 $$G = 1 + \left( {\frac{1}{n}} \right)\_(\frac{2}{{mN^{2} }})\sum\nolimits_{{i = 1}}^{n} ( N\_i + 1)y_{i}$$

In the equation, *i* = 1, 2, 3 …, *n* represents the study area, *Y*_*i*_ represents the income of group *i*, and *i* is arranged in a non-decreasing order: *Y*_*i*_ ≤ *Y*_*i*_ + 1. The Gini coefficient is most sensitive to the middle-income gap.

Although there are no internationally defined standard cut-off values, it is commonly recognized that a Gini index < 0.2 corresponds to perfect income equality, 0.2–0.3 corresponds to relative equality, 0.3–0.4 corresponds to relatively reasonable income gap, 0.4–0.5 corresponds to high income disparity, and a value above 0.5 corresponds to severe income disparity [24, 25].

In this study, the Gini coefficient was used to quantitatively reflect the equity of MRI distribution, based on the area population and the MRI configuration. We applied the coefficient to analyze and compare the equity in each sample city. STATA 15.0 software was used to conduct the analysis. STATA15.0 with the Distributive Analysis Stata Package (DASP) was used to calculate the Gini coefficient and concentration index. The primary objective of DASP is to facilitate distributive analysis using Stata for researchers and policy analysts.

### Agglomeration degree

The Health Resources Agglomeration Degree (HRAD) is the proportion of health resources in a sub-area that accounts for 1% of the total geographical area (%). It reflects the concentration of health resources in a region [26].

The HRAD is calculated as follows:

2 $${HRAD}_{i}=\frac{{HR}_{i}/{HR}_{n}*100\mathrm{\%}}{{A}_{i}/{A}_{n}*100\mathrm{\%}}=\frac{{HR}_{i}/{A}_{i}}{{HR}_{n}/{A}_{n}}$$

*HR*_*i*_ is the quantity of a specific health resource type in a particular sub-area, and *HR*_*n*_ is the area’s total public health resources. *A*_*i*_ is the geographical area of the sub-area, and *A*_*n*_ is the total size of the geographical area.

The population agglomeration degree (PAD) is the proportion of the population (%) in a sub-area that accounts for 1% of the total area size; it reflects the degree of population agglomeration relative to the total population [27].

3 $${PAD}_{i}=\frac{{P}_{i}/{P}_{n}100\mathrm{\%}}{{A}_{i}/{A}_{n}*100\mathrm{\%}}=\frac{{P}_{i}/{A}_{i}}{{P}_{n}/{A}_{n}}.$$

Here, *P*_*i*_ is the population of the sub-area, and *P*_*n*_ is the total population of the area.

It is generally believed that an HRAD value approaching 1 represents a more equitable allocation of healthcare resources based on geographical area. When the HRAD is above 1, health resource allocation by geography is redundant in that area. A ratio of HRAD/PAD = 1—that is, the proportion of health resources in a sub-area that accounts for 1% of the total geographical area relative to the proportion of the population in the same sub-area is equal—indicates a state of absolute equity in health resource allocation. Moreover, a HRAD/PAD ratio > 1 indicates that the health resources are sufficient for the area’s population, while a HRAD/PAD ratio < 1 indicates that allocation is insufficient [28–30].

### Data envelopment analysis

Data envelopment analysis is a fixed frontier non-parametric method that does not require the setting of a production function and is suitable for measuring the performance and relative efficiency of a decision-making unit. Furthermore, DEA effectively delineates the efficient frontier (EF) of a production possibility set (PPS). The EF represents the maximal output attainable from each input level [31]. Its economic interpretation of validity is based on the microeconomic production function y = f (x). When the point (x, y) satisfies y = f (x), it is located on the surface of the production function, which is defined as “technical efficiency.” (Figs. 1, 2).

**Fig.3:**
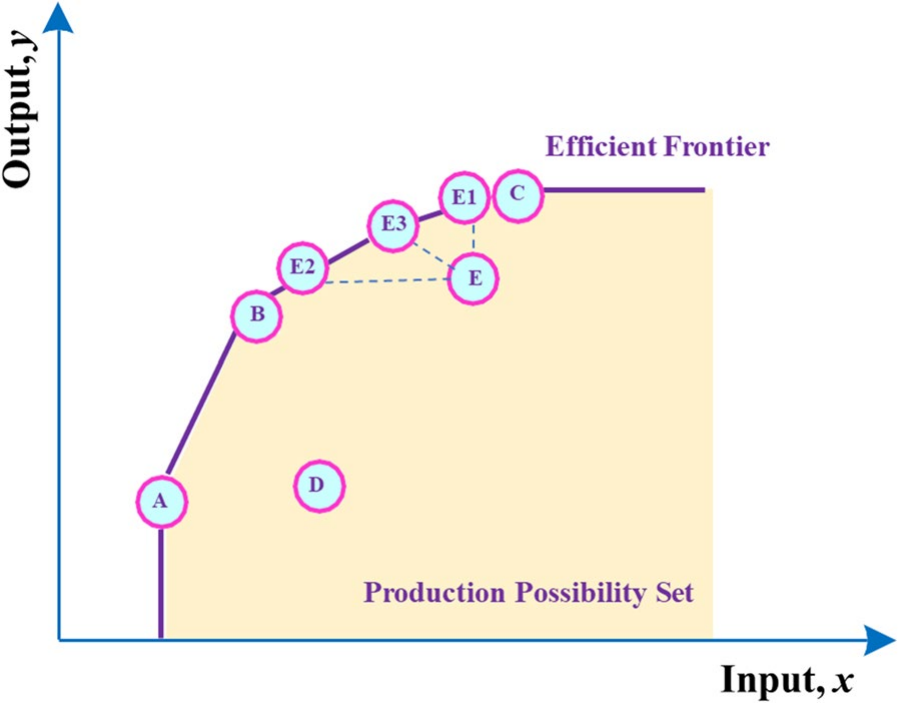
Graphical presentation of the DEA approach for performance evaluation of DMUs

Figure 3 illustrates the EF and PPS, where one input and one output are considered. Based on the DEA approach, the DMUs A, B, C are technically efficient, whereas the DMUs D, E are technically inefficient in Fig. 3 [31]. While inefficient PPS E can improve its relative efficiency in several ways: increase output without changing its original input (move to E1), use fewer inputs to produce the same level of outputs (E2), or adjust both inputs and outputs in such a way as to reach the frontier (E3).

DEA has two models—the Charles–Cooper–Rhodes (CCR) or constant return to scale (CRS) model and the Banker–Charnes–Cooper (BCC) or variable return to scale (VRS) model. The CRS model measures the overall efficiency of each decision-making unit to determine whether its scale and efficiency are effective simultaneously. In the VRS model, the assumption of CRS is changed to that of VRS. The efficiency of decision-making units under a VRS condition is measured by dividing total efficiency into pure technical efficiency and scale efficiency [32–34]. It is simultaneously assumed that the scale return of each decision-making unit could be increasing, decreasing, or unchanged. Considering that the production technology of the health system is VRS, this study adopts the VRS model by using Eq. (4) [35, 36]:

4 $$Eff={Max}_{{u}_{r}{r}_{j0}}{\sum }_{r}{u}_{r}{y}_{r{j}_{0}}+{u}_{0}$$

s.t.

$$\sum_{r}{u}_{r}{y}_{rj}-\sum_{i}{v}_{i}{x}_{ij}+{u}_{0}\le 0$$

$$\mathop \sum \limits_{i} v_{i} x_{{ij_{0} }} = 1~~~~~~~~~u_{r} ,v_{i} \ge 0$$

where.${y}_{rj}$ is the amount of output r produced by hospital j${x}_{ij}$ is the amount of input i used by hospital j${u}_{r}$ is the weight given to the output r, (r = 1,… and t is the number of outputs)${v}_{i}$ is the weight given to the input i, (i = 1,…m and m is the number of inputs)n is the number of hospitalsj_0 is_ the hospital under assessment

We selected the MRI technology efficiency evaluation input and output indicators, which focus on the core input factors, such as people, finance, and materials, and the main output factors, such as service volume and economic income, in the MRI configuration process (Table 1) [37]. The sample data of 78 MRI facilities (of the full sample of 417) in 11 cities were collected using the “MRI Service System Evaluation” survey.

**Table 1:** Input and output index in the DEA model

Category	Variable	Unit	Definition
Input	Human resources	person	Number of doctors, nurses, technicians, and engineers in the MRI department
	MRI^a^ quantity	Set	Total MRI machines in MRI departments
	Cost/year	Million USD	Total cost per year, including equipment depreciation cost, annual human resource cost, equipment maintenance cost, depreciation cost of buildings, and other miscellaneous expenses
Output	Annual MRI patient number	Number	Total patient volume for MRI examinations, including the number of outpatients and inpatients, and the number of physical examinations
	Annual income	Million USD	Income from MRI examinations, including inpatient, outpatient, and physical

^a^*MRI* magnetic resonance imaging

DEAP 2.1, a tool developed to analyze efficiency, was used to conduct the DEA analysis.

### MRI configuration geographic information display

ArcGis geographic information software was used to present the MRI geographical allocation. The map of Henan province is derived from the national basic geographic information.

## Results

### MRI allocation status

Table 2 shows that there were 417 MRI machines in the 11 sample cities in 2017, of which 16.07% are 3.0 T (scientific research level), 33.57% are 1.5 T (clinical research level), and 50.36% are 1.0 T (clinical practical level). We used the first letters of the cities’ full names as codes for the sample. The provincial capital, Zheng Zhou, has the most installations—by both population and geographical sizes—among all the sample cities. The average increase rate during the last 10 years is 20.23% (Table 2).

**Table 2:** MRI distribution in prefecture-level cities of Henan province (2017)

City	MRI Allocation by quantity				MRI allocation		AGR (%) (2008–2017) %
	Total	Scientific research (3.0 T)	Clinical research (1.5 T)	Clinical practical (≤ 1.0 T)	Per million population	Per thousand square kilometers	
An Yang	32	6	13	13	6.24	5.71	14.28
Ji Zhou	18	2	7	9	5.06	4.42	18.04
Kai Feng	21	2	8	11	4.38	3.26	39.55
Nan Yang	56	5	20	31	5.45	2.11	5.92
San MenXia	16	0	6	10	7.05	1.55	44.97
Shang Qiu	40	2	14	24	5.48	3.74	19.95
Xin Xiang	36	6	15	15	6.24	4.35	11.14
Xin Yang	23	1	14	8	3.56	1.22	37.97
Xu Chang	25	3	7	15	5.67	5.00	34.66
Zheng Zhou	95	38	26	31	8.07	12.75	12.46
Zhou Kou	55	2	10	43	6.28	4.58	46.96
Total	417	67	140	210	5.91	3.62	20.23

**Fig. 4:**
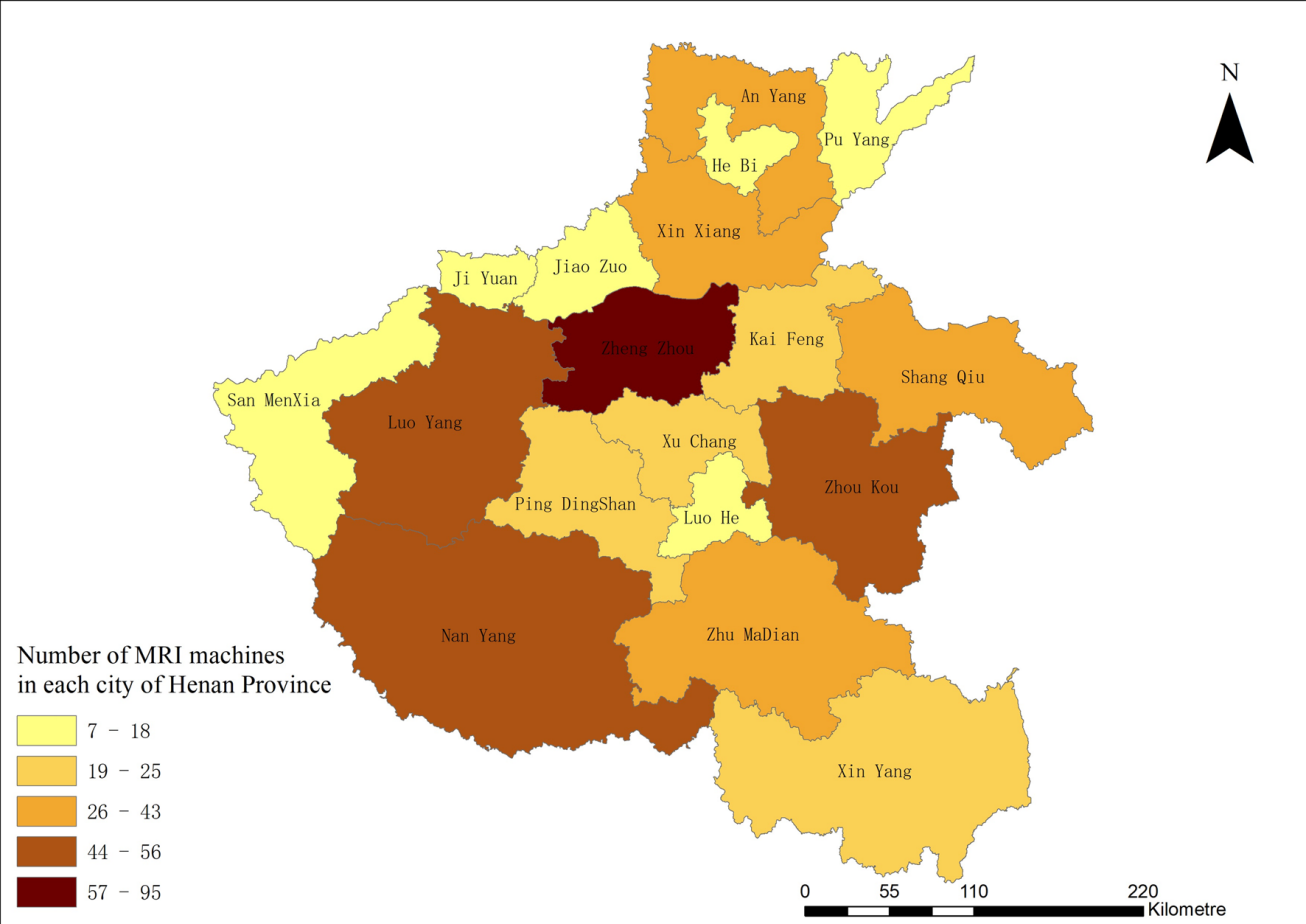
MRI distribution by volume in 11 prefecture-level cities of Henan province

Figure 4 shows the absolute number of MRI machines deployed in prefecture-level cities in Henan province. Through GIS, it is observed that Zheng Zhou, as the provincial capital, has the highest configuration number.

**Fig.5:**
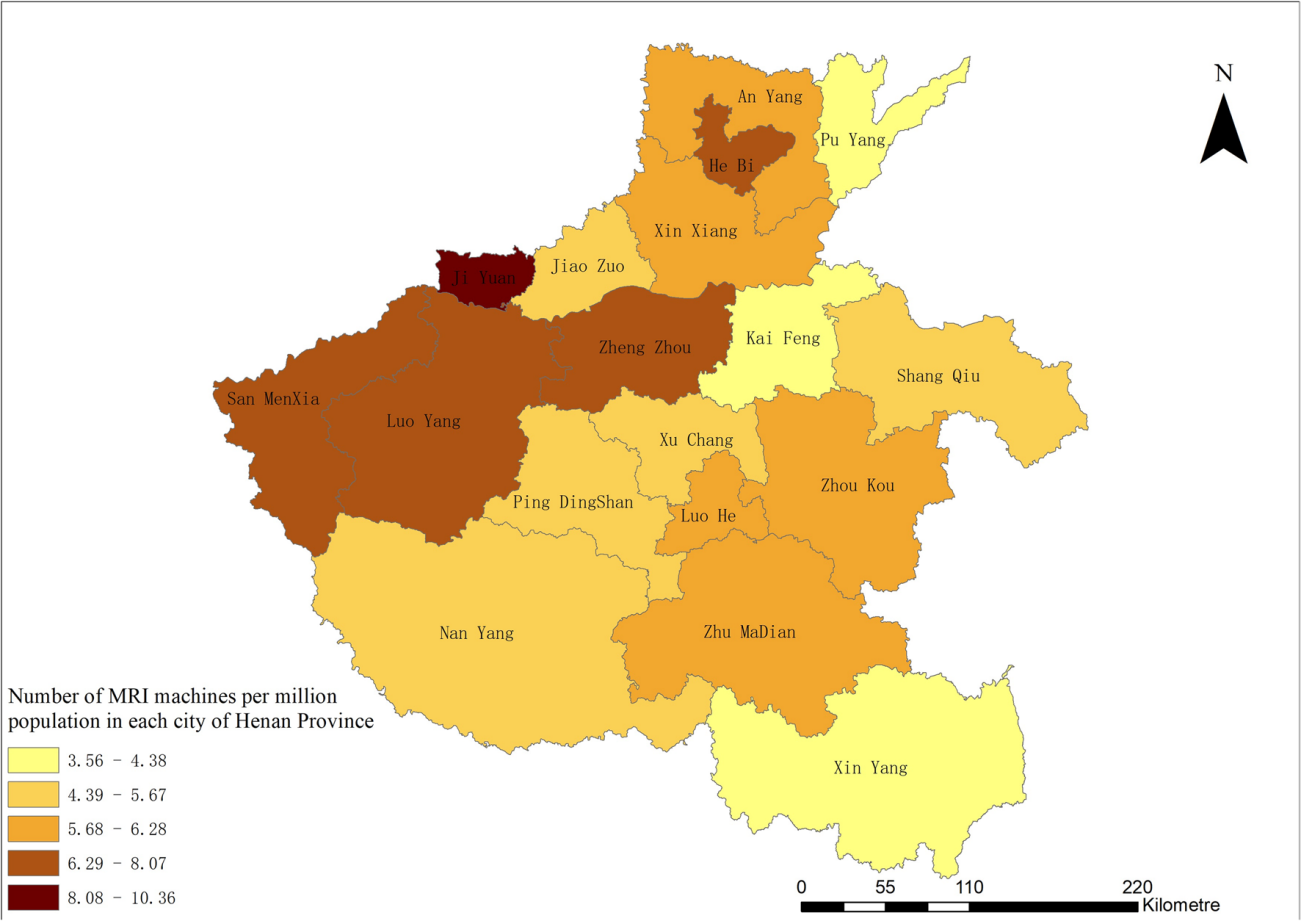
Number of MRI machines per million population in each city of Henan province

Figure 5 shows the MRI configuration per million population. We observe a clear imbalance in city-based MRS configuration. The number of MRI machines per million population in Zheng Zhou, Luo Yang, and San MenXia is the highest (approximately 6–8); however, in Xin Yang, Kai Feng, and Pu Yang, the allocation is among the lowest in the province, with fewer than 4.5 machines per million population.

### Equity assessment and agglomeration degree analysis in the 11 sample cities

The 18 prefecture-level cities in the Henan province had an overall Gini coefficient of 0.1190 in terms of MRI configuration, while the coefficient of the 11 sample cities was 0.117, showing a relatively equal configuration status at the provincial level. However, the equity within each of the cities showed a remarkable difference.

By amalgamating the Gini coefficient and agglomeration indicators, it was determined that the Gini coefficient values of MRI allocation in Shang Qiu, Xin Yang, and Xu Chang reflected a moderately equitable distribution (0.292, 0.260, and 0.256, respectively). Among them, Shang Qiu and Xu Chang demonstrated HARD/PAD ratios less than 1, signifying a marginal inadequacy in the accessibility of population-based resource allocation (0.915 and 0.946, respectively). In contrast, Xin Yang showed considerably lower resource allocation (0.595). Regarding MRI allocation equity, Jiao Zuo, Nan Yang, Xin Yang and Zhou Kou demonstrated reasonable levels with Gini coefficient values ranging from 0.30 to 0.40 (0.370, 0.325, 0.320, and 0.346, respectively); while the resource allocation of Jiao Zuo and Nan Yang exhibited some inadequacy (0.844 and 0.909, respectively), Xin Xiang and Zhou Kou had slightly surplus resource allocation (1.042 and 1.048, respectively). The Gini coefficients of An Yang, Kai Feng, San MenXia, and Zheng Zhou exceeded 0.40, signifying an evident inequality in the MRI configuration in each city (0.459, 0.493, 0.400, and 0.455, respectively). Further analysis revealed that An Yang, San MenXia, and Zheng Zhou exhibited relatively surplus resource allocation (1.729, 1.177, and 1.347, respectively), while Kai Feng presented inadequate resource allocation.

The results related to these indicators are shown in Table 3.

**Table 3:** Gini coefficient and agglomeration indicators of sample cities in 2017

Cities	Gini coefficient	Agglomeration indicators		
		PAD	MRI agglomeration	
			HRAD^a^	HRAD/PAD^b^
An Yang	0.459	0.594	1.028	1.729
Jiao Zuo	0.370	1.486	1.254	0.844
Kai Feng	0.493	1.301	0.950	0.730
Nan Yang	0.325	0.659	0.599	0.909
San MenXia	0.400	0.367	0.432	1.177
Shang Qiu	0.292	1.158	1.06	0.915
Xin Xang	0.320	1.188	1.237	1.042
Xin Yang	0.260	0.579	0.345	0.595
Xu Chang	0.256	1.499	1.419	0.946
Zheng Zhou	0.455	2.686	3.617	1.347
Zhou Kou	0.346	1.245	1.304	1.048

^a^*HRAD* Health resources agglomeration degree

^b^*PAD* population agglomeration degree

### Efficiency assessment of MRI allocation in Henan province

We compiled efficiency data from 78 MRI machines located across 32 hospitals in 11 cities. These 78 machines constitute a fraction of the total number of machines within the city where the hospital is situated. The corresponding findings are presented in Table 4.

**Table 4:** Basic information of input–output indicators for sample cities (2017)

City	Input indicators			Output indicators	
	Human resource	MRI Qty	Cost/year (million USD)	Annual MRI patient number	Annual income (million USD)
		(set)			
An Yang	24	3	1.19	28,147	3.15
Jiao Zuo	40	4	1.28	34,857	3.52
Kai Feng	40	10	1.05	45,541	3.28
Nan Yang	29	3	1.05	34,169	3.54
San MenXia	23	3	1.05	20,120	1.76
Shang Qiu	28	5	1.41	66,141	4.04
Xin Xiang	36	6	4.64	81,220	9.16
Xin Yang	21	2	1.47	22,666	2.79
Xu Chang	18	3	0.80	17,007	1.43
Zheng Zhou	195	34	22.31	404,496	45.04
Zhou Kou	27	5	1.65	78,330	9.25

Table 5 reveals that of the 11 cities, Zhou Kou is the only one that attained comprehensive efficiency, whereas the remaining 10 cities demonstrated inefficiency. Despite exhibiting a pure technical efficiency score of 1, Kai Feng, Nan Yang, Shang Qiu, Xing Yang, Xu Chang, and Zheng Zhou displayed scale efficiencies lower than 1, implying that their inefficiency could be attributed to the scale of their MRI-related resource input. Among those cities, Zheng Zhou is the only one with a diminishing scale return, which suggests an over-deployment of its MRI-related resources. In contrast, An Yang, Jiao Zuo, San MenXia, and Xin Xiang displayed pure technical efficiency and a scale return below 1, signifying that the resource input is not optimally utilized, and the configuration does not achieve the optimal scale.

**Table 5:** Efficiency of MRI utilization in sample cities (2017)

City	Crest^a^	Vrest^b^	Scale	Return to scale
An Yang	0.599	0.869	0.689	irs
Jiao Zuo	0.576	0.674	0.855	irs
Kai Feng	0.912	1	0.912	irs
Nan Yang	0.727	1	0.727	irs
San MenXia	0.428	0.714	0.600	irs
Shang Qiu	0.990	1	0.990	irs
Xing Xiang	0.864	0.907	0.953	drs
Xin Yang	0.753	1	0.753	irs
Xu Chang	0.447	1	0.447	irs
Zheng Zhou	0.759	1	0.759	drs
Zhou Kou	1	1	1	–
Mean	0.732	0.924	0.790	

Scale = scale efficiency = Crest/Vrest

^a^*Crest* technical efficiency from CRS DEA

^b^*Vrest* technical efficiency from VRS DEA

### Equity-efficiency matrix analysis

**Fig.6:**
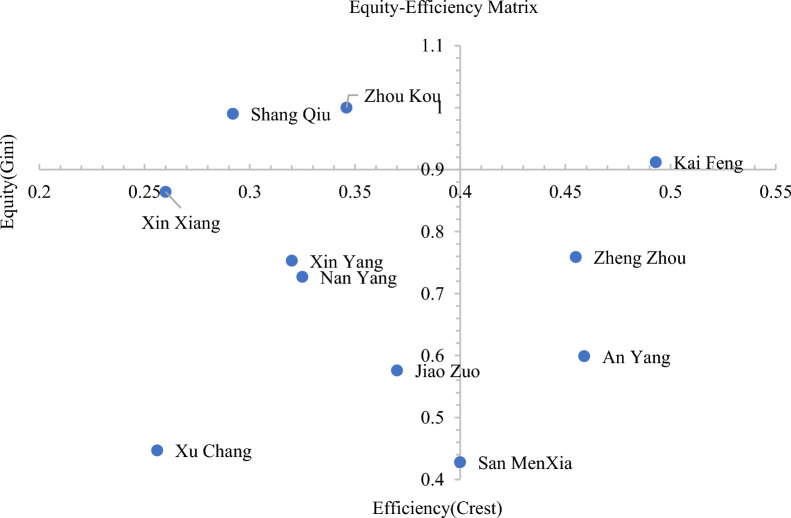
Matrix diagram of Gini coefficient and Crest in 11 sample cities in Henan province

We used the equity-efficiency matrix to delve deeper into the equity and efficiency level in all sample cities. Figure 6 clearly shows that only Shang Qiu and Zhou Kou achieved relatively optimal resource utilization. Although equity was relatively fair for cities such as Xin Xiang, Nan Yang, Jiao Zuo, and Xu Chang, the efficiency level remained low. However, for Zheng Zhou, An Yang, and San MenXia, both equity and efficiency were much lower than those of other cities. Despite being the provincial capital and having the most aggregated resources, Zheng Zhou’s equity and resource efficiency lagged behind most other cities in the sample.

## Discussion

The configuration number of MRI machines in China exceeded 6000 sets in 2017, indicating a high growth rate since MRI was introduced in China in 1985 [38]. In the late twentieth century, poor management and configuration led to a race for large medical equipment in hospitals. Following the implementation of the Management Policy on Configuration and Utilization of large medical equipment along with the CON policy, the situation has improved, with a more modest increase observed in the number of MRI configurations [3].

Henan’s MRI configuration shows the same development pattern. In 2004, the province had only 18 MRI machines; however, during the next 13 years, this increased at a rate of 30.9% per annum. The number of MRI machines per million population was 5.91, which exceeded the national average. However, MRI configuration in the province still faces several challenges.

### MRI configuration structure cannot support future development

This study’s findings suggest that the current MRI configuration structure in the province may not be able to accommodate future development, despite the average increase of 20.23% per million population over the last decade. More than 50% of these MRI machines are 1.0 T or lower, which can impede clinical application and reduce efficiency. Consequently, hospitals’ capacity for future development may be compromised, potentially resulting in a loss of patients for these hospitals. This may explain the reason underlying the high patient volume for MRI in Zheng Zhou, the provincial city, which has a five-fold or even higher patient volume than other cities. This situation could impede the acquisition of new equipment for hospitals in small cities or rural areas, as these hospitals may find it challenging to fulfill certain requirements of the CON policy, such as patient volume and the number of qualified technicians, which limit their ability to acquire new or higher-level MRIs.

### Overall MRI equity in Henan is relatively good, but variance exists within cities

To assess the impact of the management policy in Henan province, we conducted an evaluation of both equity and efficiency. Our findings indicate that the provincial MRI configuration is comparatively satisfactory, which suggests the effectiveness of provincial government intervention in managing large medical equipment allocation. Nonetheless, the analysis of equity at the municipal level revealed significant disparities because of variations in cities' geographical size, population, and economic conditions. The Gini coefficients of the sampled cities displayed substantial differences, ranging from a high of 0.256 to a low of 0.493. Furthermore, despite high equity in certain cities, the MRI volume per million population was relatively low. For some cities, such as Zheng Zhou and An Yang, the Gini coefficient and agglomeration degree indicate the worst equity among the cities surveyed. This observation is particularly noticeable in the case of Zheng Zhou, the provincial capital, where health resources are significantly concentrated; the high annual MRI patient volume may have resulted from a high volume of patients from other cities seeking medical attention. The influx of patients into Zheng Zhou suggests that patients unable to receive adequate care at local hospitals are being referred to city hospitals; especially given the more convenient transportation system, patients are increasingly willing to obtain diagnostic services at the provincial-level hospitals. This trend is contrary to the fundamental principle of the hierarchical diagnosis and treatment system. The influx of patients increased higher MRI allocation in provincial-level hospitals in Zhengzhou, further exacerbating the inequities. The most probable factor causing municipal-level inequity is the quotas being solely based on provincial-level information, with no consideration for municipal-level data. Therefore, quota formulation should include an evaluation of equity within individual cities to mitigate such disparities.

### Overall MRI utilization is ineffective and scale efficiency varies among cities

The overall efficiency of MRI utilization across the 11 cities was 0.732, with the primary contributing factor being inefficiencies of scale. This highlights the need for further optimization in resource allocation among the cities to address the existing imbalances. The findings demonstrate that increasing and deploying resources in most sample cities could improve both scale and overall efficiency. Excessive resource concentration in Zheng Zhou has resulted in low scale efficiency and hindered access to healthcare for patients residing in closer proximity to other hospitals, which is contrary to the primary objective of healthcare reform.

### Indicators of equity and efficiency should be dynamically monitored by provincial health departments during policy execution

Unlike prior research in the field, this study focuses on municipal-level configuration. To enhance equity and efficiency, it is imperative to consider factors such as equity, allocation volume, and efficiency of MRI configurations in various cities on an individual basis. The fundamental objective of governmental intervention in resource allocation is to ensure equitable and efficient distribution, which are interdependent. To provide precise guidance for optimizing provincial-level allocation, provincial health departments must possess comprehensive MRI data, encompassing both equity and efficiency metrics, for each city [39–43].

Currently, large medical equipment is allocated based on the principle of equity and optimal allocation; however, the observed allocation patterns reveal an obvious aggregation effect in provincial capital cities like Zheng Zhou, primarily because of the concentration of patients and medical resources. Although the guidelines specify the requirements for the standardized use of MRI equipment, the policy lacks a monitoring and feedback mechanism to gauge the efficiency of utilization. Furthermore, an irrational equipment composition could potentially cause underutilization [44]. The comprehensive efficiency of MRIs in Henan is below 1. Our results reveal that out of all sample cities, only one city achieved comprehensive efficiency, indicating an overall ineffectiveness in municipal-level MRI utilization.

Liu and Chen present a similar conclusion [45]. The existing policy prioritizes equitable distribution, while insufficient attention is devoted to an appropriate mechanism for monitoring provincial and municipal utilization. To facilitate future optimization of resource allocation, it is imperative to establish a dynamic equity and efficiency data collection mechanism that captures both equity and efficiency metrics at municipal-level hospitals.

### Provincial-level MRI configuration plan should be further optimized

Based on the analysis of the equity, HARD, and efficiency of various cities in Henan province, we found that Zhou Kou has the best equity, resource allocation and efficiency among the sample cities in Henan. Among all the sample cities, Kai Feng needs to increase MRI equipment the most, as its resource allocation is insufficient, but the efficiency of the existing equipment is relatively high. However, there is a large gap in its allocation fairness, and adding new equipment can improve accessibility for patients. The equity of allocation in Jiao Zuo, Shang Qiu, Xin Yang, and Xu Chang is relatively reasonable and fair, but their resources are somewhat insufficient. By increasing the allocation, the scale efficiency can be increased. Therefore, it is possible to consider increasing the allocation appropriately if the equipment efficiency is improved. The resource allocations of San MenXia, Anyang, and Zhengzhou are redundant, and there is a significant gap in allocation fairness. Meanwhile, the equipment efficiency is low. Therefore, it is necessary to focus on improving efficiency first and then increase the allocation to improve fairness and scale efficiency. Especially in Zhengzhou, by enhancing the medical service capacity in surrounding cities, the hierarchical diagnosis and treatment mechanism should be used as much as possible to divert patients and avoid further aggregation in provincial hospitals. Xinxiang shows good allocation fairness, but the resource allocation is slightly redundant. As the scale efficiency will decrease as equipment increases, it is essential to improve equipment utilization efficiency.

This study aims to determine the MRI configuration improvement priority on a municipal level, based on cities’ equity and efficiency information. Furthermore, its objective is to establish an MRI configuration improvement plan at a provincial level by comparing the equity and efficiency of provincial cities. To summarize, healthcare resource allocation should first be equitable, while considering efficiency, and the target should be continuously adjusted according to different policy implementation stages and different effects of equity and efficiency. This study also aims to provide a more scientific method for the provincial health administrative department to deploy MRI machines at a municipal level. The configuration and management policies of large medical equipment should be adjusted continuously, according to the results of the implementation phases, based on the equity and efficiency of configuration. Lastly, it should also optimize the configuration structure to foster effective resource use, thereby achieving the goal of government intervention.

## Conclusions

Henan province in China has a population of nearly 10 million and serves as a case study for evaluating the crucial equity and efficiency of MRI configuration. Our findings provide valuable evidence-based insights that could aid in the continual scientific and dynamic adjustment of provincial-level CON policies in Henan province. They may also serve as a guide for optimizing CON policies for other large medical equipment. Moreover, our study offers a useful research perspective for other rapidly developing countries seeking to establish a scientific management system for large medical equipment.

## Limitations

Despite providing numerous valuable insights, this study is subject to certain limitations. First, the analysis is based on a sample of 11 out of the 18 cities in Henan province; comprehensive data on MRI deployment and utilization in the entire province was unavailable. Second, the efficiency analysis is constrained at the municipal level because of the limited sample size. Therefore, further analysis into MRI utilization, such as efficiency by hospital level or hospital attribute (public or private), was not feasible within the scope of this study. Third, this study is based on static cross-sectional data, which limits dynamic factors that may influence patients’ habits and behavior of diagnosis and treatment. For instance, the improvement of transportation infrastructure, which may facilitate greater movement of patients between cities, and the tendency of small-city patients to seek treatment in large city hospitals, were not investigated owing to a lack of data. Consequently, equipment utilization efficiency may have some bias and may not reflect the actual MRI utilization efficiency. A further limitation of this study is that the DEA analysis is based on a sample of hospitals rather than the entire population, thereby restricting our ability to develop a quantitative optimization plan for MRI configuration. Future research on this topic would benefit from a more comprehensive consideration of dynamic factors that affect equipment utilization efficiency, such as those mentioned earlier, by utilizing panel data. This approach would facilitate a more accurate and in-depth calculation of equipment utilization efficiency, thereby providing more robust insights for policymakers and healthcare professionals. Finally, developing a comprehensive evaluation system for MRI resource allocation and relevant indicators is imperative to systematically assess the effectiveness and impact of large medical equipment configuration management policies.

## Figures and Tables

**Table 6:**
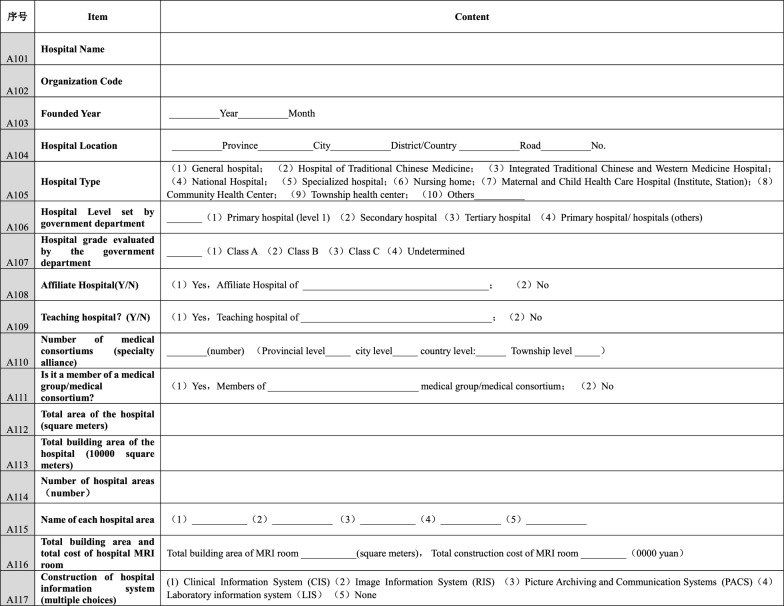
Hospital Information

Instructions for filling in the form: (1) It is recommended that the medical department (department) and hospital office assist in filling in the form; (2) The building area and total cost of MRI room can be estimated if it cannot be accurately calculated for a long time; (2) Fill in the basic information of your hospital as of December 31, 2017; (3) Please complete and upload the form within three weeks after receiving it; (4) The number of medical consortiums refers to the total number of medical consortiums in all departments established or participated in by the hospital.

**Table 7:**
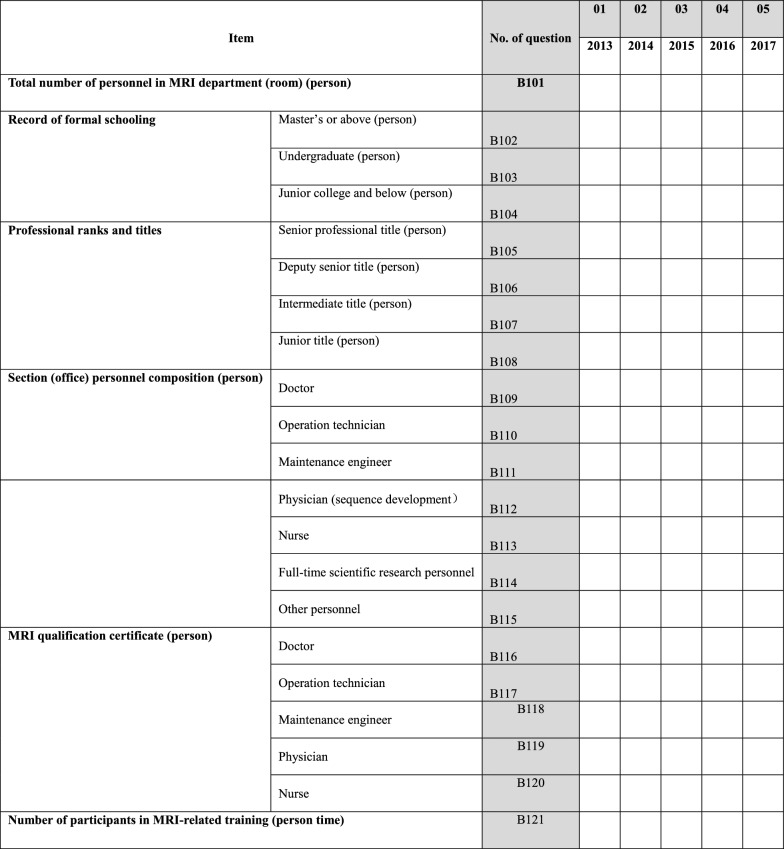
MRI department (room) staffing questionnaire

Instructions for filling in the form: (1) This form is recommended to be filled in by the department head or secretary; (2) The statistical time is as of December 31 every year. If there are no corresponding categories of personnel in some forms, please write 0; (3) The number of participants in MRI-related training refers to various related trainings organized by MRI manufacturers; (4) If the MRI department (office) is not independent, the composition of the staff of the MRI department (office) shall be calculated by the director of the department according to the actual situation of MRI shift scheduling; (5) Fill in the information from 2013 to 2017 in the form. For hospitals operating after January 2013, please fill in the relevant information from the month in which the hospital began to operate to December 31 of that year; (5) Please complete and upload the form within 2 weeks after receiving it.

**Table 8:**
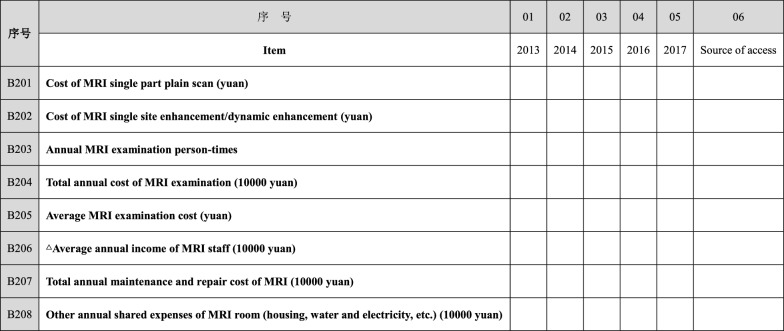
MRI department (room) expense information questionnaire

Instructions for filling in the table: (1) The average MRI examination cost is the total annual MRI examination cost/MRI annual examination person time; (2) (△) If it is impossible to separate the average annual income of employees in the MRI room (the average annual income of employees includes basic salary, allowance allowance, bonus, social security payment, food allowance, performance salary, etc.), please estimate and indicate in the column of access; (3) The table data shall be indicated to two decimal places. (4) Please fill in the information from 2013 to 2017. For hospitals operating after January 2013, please fill in the relevant information from the month in which the hospital began to operate to December 31 of that year; (5) Please fill in the last column of the table according to the following classification number for the data access way filled in this row: ① HIS/PACS system, ② manual recording, ③ estimation, ④ others (if any, please specify the specific situation); (6) This form is suggested to be filled by the department head or secretary; (7) Please complete and upload the form within 2 weeks after receiving it.

**Table 9:**
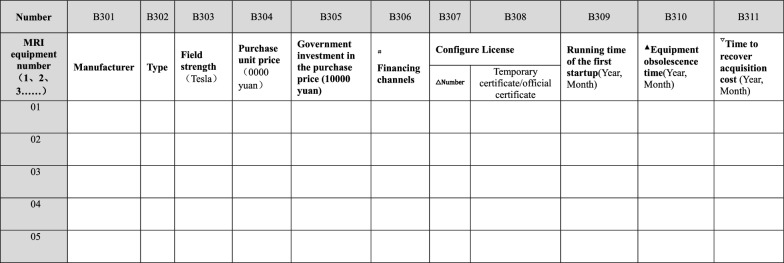
Hospital MRI equipment configuration questionnaire

Instructions for filling in the form: (1) The time filled in the form should be accurate to month; (2) # Financing channels include: ① purchase with self-raised funds of the hospital, ② purchase with joint investment of the government and hospital, ③ lease, ④ enterprise investment, ⑤ financing, ⑥ acceptance of donations, ⑦ others; (3) Configuration license (△) number: if approved, write “Configuration license number”; if not approved, write “No”; (4) (▲) Equipment obsolescence time: write the year and month of obsolescence if it has been eliminated, and write “No” if it has not been eliminated; (5) (▽) Time to recover the acquisition cost: if the cost is not recovered temporarily, please fill in the estimated time to recover the cost; (7) The amount shall be filled in with the assistance of the Finance Department (Section) or the Equipment Department. If the data cannot be obtained, please estimate and indicate; (8) This table includes: MRI equipment used (including obsolete) and in use in the hospital from January 2013 to December 2017; (9) Please fill in the information of all MRI equipment configured by the hospital. For units with two or more MRI equipment, the equipment number should be consistent with the equipment number in other tables; (10) This form is recommended to be filled by the department head or secretary; (11) Please complete and upload the form within 2 weeks after receiving it.

## Data Availability

Data were collected from the 2018 statistical yearbook of Henan province.

## Abbreviations

AGR: Annual growth rate
CON: Certificate of Need
CRS: Constant return to scale
DEA: Data envelopment analysis
HRAD: Health Resources Agglomeration Degree
MRI: Magnetic resonance imaging
NHC: National Health Commission
PAD: Population agglomeration degree
VRS: Variable return to scale
